# Low-dose radiotherapy of osteoarthritis: from biological findings to clinical effects—challenges for future studies

**DOI:** 10.1007/s00066-022-02038-6

**Published:** 2023-01-05

**Authors:** Thomas Weissmann, Michael Rückert, Florian Putz, Anna-Jasmina Donaubauer, Markus Hecht, Sören Schnellhardt, Philipp Schubert, Johannes Roesch, Daniel Höfler, Oliver J. Ott, Marlen Haderlein, Sebastian Lettmaier, Rainer Fietkau, Benjamin Frey, Udo S. Gaipl, Lisa Deloch

**Affiliations:** 1 Department of Radiation Oncology, Universitätsklinikum Erlangen, Friedrich-Alexander-Universität Erlangen-Nürnberg, Erlangen, Germany; 2 Translational Radiobiology, Department of Radiation Oncology, Universitätsklinikum Erlangen, Friedrich-Alexander-Universität Erlangen-Nürnberg, Erlangen, Germany; 3 Comprehensive Cancer Center Erlangen-EMN, Erlangen, Germany

**Keywords:** Osteoimmunology, Inflammation, Benign diseases, Translational research, Clinical application

## Abstract

Osteoarthritis (OA) is one of the most common and socioeconomically relevant diseases, with rising incidence and prevalence especially with regard to an ageing population in the Western world. Over the decades, the scientific perception of OA has shifted from a simple degeneration of cartilage and bone to a multifactorial disease involving various cell types and immunomodulatory factors. Despite a wide range of conventional treatment modalities available, a significant proportion of patients remain treatment refractory. Low-dose radiotherapy (LDRT) has been used for decades in the treatment of patients with inflammatory and/or degenerative diseases and has proven a viable option even in cohorts of patients with a rather poor prognosis. While its justification mainly derives from a vast body of empirical evidence, prospective randomized trials have until now failed to prove the effectiveness of LDRT. Nevertheless, over the decades, adaptions of LDRT treatment modalities have evolved using lower dosages with establishment of different treatment schedules for which definitive clinical proof is still pending. Preclinical research has revealed that the immune system is modulated by LDRT and very recently osteoimmunological mechanisms have been described. Future studies and investigations further elucidating the underlying mechanisms are an essential key to clarify the optimal patient stratification and treatment procedure, considering the patients’ inflammatory status, age, and sex. The present review aims not only to present clinical and preclinical knowledge about the mechanistic and beneficial effects of LDRT, but also to emphasize topics that will need to be addressed in future studies. Further, a concise overview of the current status of the underlying radiobiological knowledge of LDRT for clinicians is given, while seeking to stimulate further translational research.

## Osteoarthritis

Osteoarthritis (OA) accounts for most cases of degenerative joint disease and carries a significant socioeconomic burden, consuming up to 1.0–2.5% of the gross domestic product in the Western world [1]. The etiology of the disease is multifactorial, encompassing multiple genetic, biological, and biochemical risk factors such as female gender, advanced age, obesity, or mechanical wear and tear [2]. Due to ongoing scientific work, the view of the pathogenesis has changed tremendously, from simple mechanical cartilage degradation to a complex interaction of different types of tissues and cells, with a significant role played by the immune system [3].

Although OA already accounts for approximately 15% of all musculoskeletal consultations in primary care, rising case numbers have to be expected as a result of an aging population and the prevalence and incidence of OA in this age group [4]. Treatment options in general range from conservative measures like physical therapy, oral, or topical drug treatment, to the application of injections using corticoids or hyaluronic acid [5–7]. Further, more invasive joint-preserving measures or even joint replacement are viable options for advanced degrees of impairment and conservatively unmanageable symptomatic burden [8, 9]. The direct costs deriving from treatment itself as well as indirect costs arising from the need for rehabilitation or resulting from invalidation and absence from work add up to tremendous costs of up to several billions of Euros each year. Sources have reported losses of up to 70,000 working years and over 10,000,000 working days per year due to invalidation annually. The high prevalence of comorbidities in higher age groups mostly affected by OA limits the scope for oral medication, risking renal impairment or gastric ulcerations, for example [10]. Patients in advanced ages often additionally present in a reduced physical condition and with comorbidities, creating higher risks for surgical complications and often requiring prolonged medical support postoperatively, ultimately resulting in higher costs of overall treatment [11, 12]. Therefore, novel and complementary therapies are urgently needed for OA.

## Low-dose radiotherapy

Low-dose radiotherapy (LDRT) has been successfully used for several decades in the treatment of inflammatory and degenerative diseases based on convincing clinical outcomes [13]. However, the underlying radiobiological mechanisms have been neglected and thus remained unclear for a long period of time, as is also the case for large randomized studies.

While the general perception in radiotherapy claims higher doses to have superior treatment effect, studies carried out in the field of LDRT have shown lower dosages to be equivalent or even more beneficial in the treatment of OA. Ott et al., who carried out a randomized clinical trial comparing single doses of 0.5 vs. 1.0 Gy, revealed that there is no additional treatment benefit to be obtained from higher doses and according to the ALARA (as low as possible as high as reasonable) principle, higher doses should therefore be abandoned in this context. A single dose per fraction of 0.5 Gy should thus be viewed as the standard single dose to be used for the treatment of OA according to current knowledge [14].

In contrast to the absolute value of the single dose, temporal aspects of the treatment schedule (i.e., applying radiation twice, three times, or even more frequently every day) have not been sufficiently investigated in the past and more research needs to be carried out in this regard to obtain scientific evidence.

Another issue that has to be addressed in the future is the optimal time interval for application of a possible second or even third or fourth irradiation series. In clinical routine, the application of a second series is usually very well accepted, whereas the indication for the application of a third or fourth series is rather frankly indicated and applied. Optimal timespans between different series are unclear as well. In clinical daily use, the application of a second series 10–12 weeks after initial irradiation is very well accepted, while a third series is mostly applied 6 months later. The use of a fourth series is mainly indicated on an individual basis or upon personal request. Mechanisms observed for a delayed response to a second irradiation series of LDRT after initial lack of response to a first series need to be subject to further studies in the future as well. While positive results following a third irradiation series have been reported, the application of a fourth series is rather scarce, and its potential is widely unknown [15]. Thus, these questions should be the subject of future prospective trials carrying out appropriate experiments both in and ex vivo.

## Risk factors of LDRT

Whereas in general the ionizing radiation-associated induction of hematological malignancies like leukemia or lymphoma seems to peak 6–8 years after radiation, the induction of solid malignancies seems to occur with a delay of several years [16, 17]. With target tissue mainly being bradytrophic and probably less prone to development of secondary malignancies, few to no data have been published reporting secondary malignancies following LDRT. While LDRT is currently used primarily in older patients, Weissmann et al. have shown that LDRT can achieve better results than previously thought in younger age groups, questioning the limitation of its use to exclusively older patient cohorts, especially regarding the low evident rates of induced malignancies in LDRT [18].

Ideal treatment options for patients suffering from OA would be expected to offer significant and long-lasting treatment effects while not requiring ongoing application for maintenance of their effect. Continuous monitoring of treatment results has shown long-lasting effects well above 2 years in prospective clinical trials [14]. Donaubauer et al. showed in an ongoing clinical trial that LDRT is capable of inducing long-lasting anti-inflammatory immunomodulatory effects in patients with chronic degenerative and inflammatory diseases [19].

While empirical data show a higher prevalence and severity of OA in women [20], observations in prospective trials in humans as well as in biological models suggest an equivalent treatment response independent of sex, offering convincing treatment results for both genders [21].

## Current challenges

The vast majority of data supporting the efficacy of LDRT are evidence based, with several multicenter studies comprising large patient numbers justifying the use of LDRT. However, large randomized trials are still missing. While large cohorts of patients undergoing LDRT show a very promising treatment response, the lumping together of different locations for easier evaluation and presentation could blur out important information on treatment response in sublocations [21]. Donaubauer et al. and Weissmann et al. were able to demonstrate precisely these differing response rates in different joint sublocations, for example the thumb or certain small joints of the foot. Another drawback of existing studies is their mostly retrospective design and insufficient patient evaluation using the numerical rating scale and the Pannewitz score [22]. While these evaluation scores are widely accepted in Germany and Eastern Europe, they have now become somewhat outdated. We recommend the use of joint-specific questionnaires in a prospective manner to improve acceptance among orthopedic and trauma surgeons.

LDRT seems to be effective when applied using a linear accelerator as well as with orthovoltage technologies. While data showing better treatment results were reported for orthovoltage therapy in clinical studies, no prospective comparative studies or biological models have been published for OA as yet, and final proof favoring either modality is still pending [21].

Again, another drawback is the lack of prospective data based on placebo-controlled randomized trials proving the effect of LDRT. It was the research of Maler and Minten investigating the effect of LDRT on OA of the hand and knee that led to one of the very few prospective sham-controlled blinded trials failing to show an effect of LDRT using state of the art questionnaires, blood sampling, and imaging for morphologic examination via ultrasound [23, 24]. Several shortcomings of these studies have been discussed and important key points proposed for the design of future prospective trials to clarify the topic. While the small number of patients limits the power of the studies, the applied fractionation does not match the currently accepted and recommended treatment schemes. The possibility of application of a second series was not considered at all. The inclusion of patients with advanced levels of OA and a long history of pain suggests selection of a highly treatment-refractory patient population past the window of opportunity for successful conservative treatment approaches [25]. A recent prospective randomized trial carried out by Niewald et al. failed to apply a truly sham-controlled group to finally clarify the topic [26].

Future studies should not only pay attention to using specific questionnaires, but also further address the validation of different imaging modalities and define clear response criteria. We believe that the main focus in setting up future successful prospective trials will have to be on unravelling the underlying molecular biological mechanisms to stratify each patient and select those patients who have the highest possibility of treatment success. One major step in this direction will be to clarify the underlying processes of inflammation and the molecular effect of LDRT to counteract this. The following section provides a basic overview of the current knowledge on the underlying mechanisms of LDRT affecting the course of OA.

## Inflammation

**Fig. 1:**
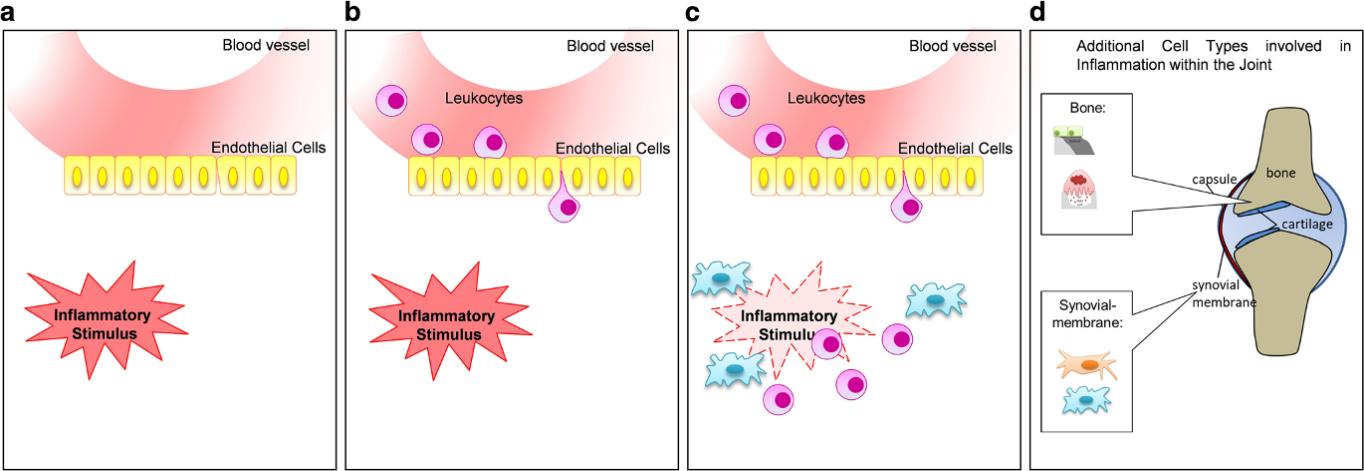
Resolution of inflammation with involvement of immune cells and additional key players within the joint. Classic inflammation is characterized by heat, redness, swelling, pain, and loss of function. Usually, if an inflammatory stimulus is induced (**a**), blood vessels become enlarged and more permeable. Further, endothelial cells alter their adhesive functions and circulating leukocytes (**b**) are able to enter the tissue. Within the tissue, the inflammation is then resolved by, e.g., immune cells (**c**). In chronic diseases such as (osteo)arthritis, the inflammatory stimulus persists and within the joint, more cells are involved (**d**). Within the synovial lining, fibroblast-like synoviocytes (FLS) and macrophages initiate and preserve the inflammatory process, which then can lead to the destruction of cartilage and bone with the involvement of osteoclasts and impairment of osteoblasts

In a healthy system, inflammatory processes are characterized by classical signs such as heat, redness, swelling, pain, and loss of function [27]. These processes are then regulated and resolved via a process that involves mediators, adhesion molecules, and cell–cell interactions [28]. In short: inflammation often occurs with infection or tissue injury. As a response, blood flow slows down, blood vessels become enlarged, and more permeable while endothelial cells (ECs) alter their adhesion properties. This enables leukocytes that are activated by local inflammatory mediators to adhere and migrate into the surrounding tissue. At the site of inflammation, leukocytes and macrophages, amongst other factors, resolve inflammation utilizing, e.g., cytokines, phagocytosis, antigen presentation, or reactive oxygen species (ROS) ([28–30]; Fig. 1a–c). In chronic inflammation of the joints, additional key players are involved. Here, macrophages and especially fibroblast-like synoviocytes (FLS), which are located in the synovium, are responsible for the initiation and upkeep of inflammation. Further, existing inflammation and mediators secreted by, e.g., FLS, are able to reduce cartilage volume and modulate bone homeostasis, thus leading to increased bone resorption and reduced bone build up, finally leading to a progressive destruction of joints [31–33]; Fig. 1d. Therefore, when investigating the biological effects of LDRT, next to classical immune cells, cells of the synovium must be considered.

## Known immunomodulatory effects of LDRT

While the underlying mechanisms behind this effectiveness are still not completely understood, it is known that LDRT-mediated mechanisms include the modulation of inflammatory processes, as described in Fig. 1; [30, 34]. The immunomodulating effects that are most likely one of the key components in LDRT effectiveness include modulation of immune cell subsets and their activation status [18, 19, 34]. As γH2AX foci, a marker for DNA damage, where found to be significantly but only mildly increased in leukocytes of patients receiving LDRT [35], cell death and DNA damage [34, 36] are also observed following LDRT; thus, additional factors, namely systemic components such as the immune system, seem to significantly contribute to the effectiveness of LDRT. While LDRT is known to affect a broad spectrum of cells, the aim of the present review is to shed light on the known effects of LDRT in clinically relevant chronic inflammatory diseases such as OA. Therefore, we will focus on results that have been obtained in an inflammatory context.

Endothelial cells (ECs) play an important role in the recruitment of leukocytes to the site of inflammation and secrete a plethora of cytokines, chemokines, and adhesion factors in their activated, inflammatory state [37–39]. It was found that after LDRT, activated ECs show a non-linear dose dependency in the regulation of secreted cytokines and their mRNA expression levels. Beneficial effects were seen especially in interleukin‑8 (IL-8), a cytokine that promotes pro-angiogenetic and anti-apoptotic effects, as well as migration of ECs into the extracellular matrix [37].

Another key role of ECs is leukocyte recruitment and attachment. Therefore, ECs were treated with tumor necrosis factor α (TNF-α) in order to mimic an inflammatory milieu before receiving LDRT. This resulted in a timely restricted decreased adhesion of peripheral blood mononuclear cells (PBMCs; i.e., leukocytes and monocytes) at 0.3–0.6 Gy that was further accompanied by a discontinuous expression of anti-inflammatory cytokine transforming growth factor(TGF)-β1 [30, 37, 40, 41].

Jurkat cells (an immortalized human T cell line) as well as peripheral blood lymphocytes (PBLs) were found to show increased adhesion to ECs following LDRT as well as alterations in ion channels and an increase in cell diameter for Jurkat cells in the low radiation dosage range [42]. Whether these observed effects result in a pro- or anti-inflammatory response needs to be further investigated, preferably in primary cell cultures in order to mimic patient status quo.

Further effects of LDRT in ECs include a reduction in inducible nitric oxide synthase (iNOS) that is responsible for a reduced EC–leukocyte interaction, as well as the production of additional anti-inflammatory cytokines such as IL-10 and decreased expression of various adhesion molecules [28]. Eckert et al. further investigated the involvement of reactive oxygen species (ROS) and anti-oxidative factors as contributors to reduced leukocyte adhesion in primary human microvascular ECs undergoing LDRT. Experiments were carried out under laminar sheer stress in contrast to static conditions, in order to mimic physiological conditions. Under laminar sheer stress, an increase in mRNA expression levels of anti-oxidative factors (0.1, 0.5, and 1.0 Gy X-rays) and a reduction of ROS (0.1 Gy X-rays) as well as reduced leukocyte adhesion (0.1 Gy X-rays) was found, while static conditions revealed no significant alterations after LDRT in a TNF-α-induced inflammatory status [43].

Another cell type that is expected to be significantly involved in the inflammation micromilieu and explicitly affected by LDRT are macrophages (MPHs). Models investigating the effect of LDRT on peritoneal MPHs isolated from BALB/c mice showed that depending on the dose, these MPHs released lower amounts of inflammatory cytokines IL-1β and TNF α [44]. Further models examined the effects of LDRT (0.01–2.0 Gy) on additionally activated peritoneal MPHs isolated from BALB/c mice. They found that viability and phagocytosis was not affected in that dose range, while migration was reduced and chemotaxis was enhanced, possibly contributing to anti-inflammatory responses. Additionally, an anti-inflammatory cytokine milieu was observed [45]. Depending on their phenotype, MPHs can carry out either rather pro- or anti-inflammatory functions; however, in the case of bone marrow-derived MPHs that were differentiated into different MPH subsets, no significant alterations in their phenotype after LDRT with doses of 0.1–2.0 Gy were found [46]. Wunderlich et al. further investigated the ability of MPHs to stimulate other immune cell types. They examined additionally activated peritoneal MPHs and cocultured them with T cells. Here, MPHs showed reduced MHCII surface expression in the dose range of 0.7–2.0 Gy. As MHCII is necessary in the response of CD4+ T cells to inflammatory stimuli, CD4+ T cells subsequently presented reduced proliferation rates potentially resulting in altered immune responses. Dendritic cells (DCs) that were coincubated with cell culture supernatants of irradiated MPHs had reduced surface levels of CD40, a costimulatory molecule necessary for cell activation, on their surface but had no impaired ability to induce CD4+ or CD8+ T cell proliferation [45]. This suggests that activated MPHs can modulate T cell-mediated immune reactions but do not have the potential to alter DC-mediated T cell responses.

Fibroblast-like synoviocytes (FLS) are key effector cells in inflammatory processes of the joints [31]. Inflammatory FLS, especially in rheumatoid arthritis, are described as being tumor like, meaning that they show a high proliferative capacity, high invasiveness, and resistance to apoptosis [47]. When treated with LDRT, however, they change this aggressive phenotype. Following LDRT, a reduction in cell number, an increase in apoptotic cells, and a reduced amount of inflammatory cytokines (IL‑6, TNF α, CXCL1) were shown by Deloch et al. [48].

Bone cells are also modulated by LDRT. Osteoblasts from inflammatory mice for example show increased mineralization at 0.5 Gy alongside an increase in osteoclast (OC) regulatory factors (osteoprotegerin) at 0.5 and 1.0 Gy [48]. Eckert et al. investigated the effects of LDRT on ex vivo differentiated OCs. No significant alterations in apoptosis were found, but total numbers of OCs were decreased. Further effects included a reduced resorbing activity, a reduced number of nuclei per OC, and a downregulation of a critical transcription factor for osteoclastogenesis (NFATc1) [49]. Similar effects were found in ex vivo differentiated OCs of an inflammatory mouse model, where OC numbers and resorptive activity were reduced in a dose range of 0.5–2.0 Gy [48].

Next to these cell type-specific effects, effects on DNA damage, cell death, and transcription factors that can, in turn, result in modulation of immune cells, were also found in response to LDRT [30, 35, 40].

## LDRT induces anti-inflammatory effects in preclinical models

**Fig. 2:**
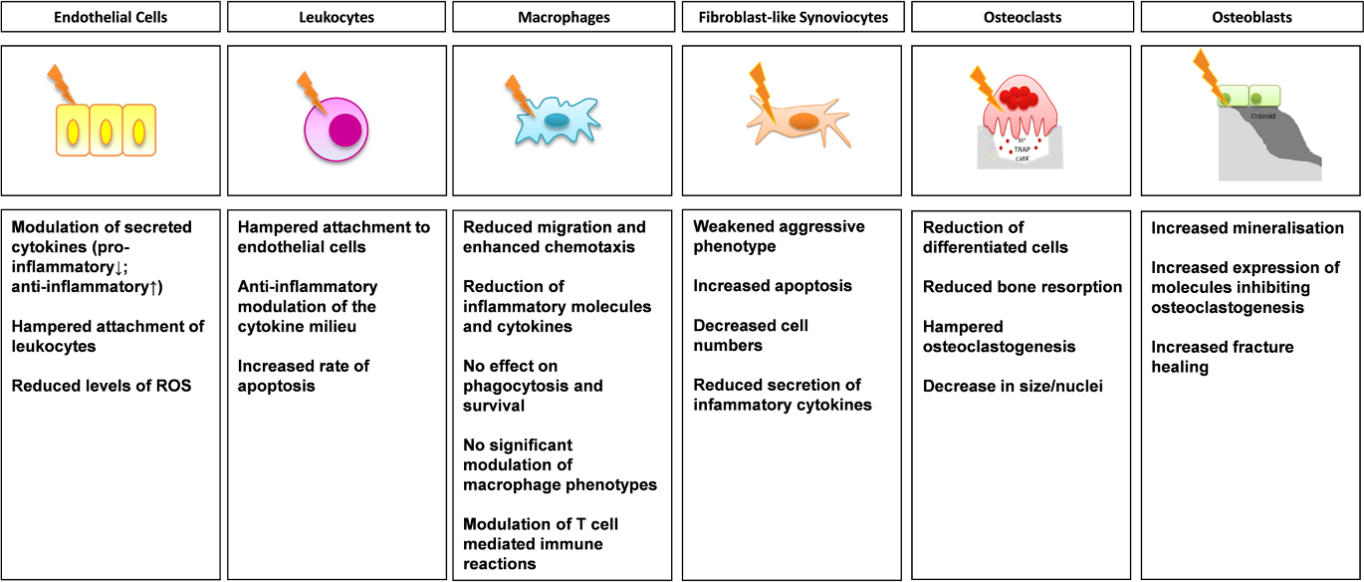
Known biological effects of low-dose-radiotherapy (LDRT; *yellow lightning bolt*) on cells involved in chronic inflammatory joint diseases. Shown are the known molecular effects after a treatment with LDRT on cells that are involved in inflammatory processes of chronic degenerative diseases of the joints. While results often vary depending on the used model and dose, an overall rather anti-inflammatory modulation of these cell types can be observed. *ROS *reactive oxygen species

Summarizing the observed ex vivo effects, while heterogenous, all contribute to a rather anti-inflammatory response (summarized in Fig. 2). The overall anti-inflammatory effects found in cell culture experiments are further supported by observations made in in vivo animal experiments.

Among the observed effects are an overexpression of anti-inflammatory TGF-β1 and decreased amounts of inflammatory cytokines and inducible heat shock protein 70 (Hsp70), resulting in an overall anti-inflammatory response. Hsp70 is a molecule that is also recognized as a danger-associated molecular pattern (DAMP), resulting in induction of inflammation. The lowest effective dose for decreased leukocyte adhesion in mouse models was found to be 0.3 Gy [28]. Abdominal irradiation of mice with 0.3 Gy after administration of an inflammatory stimulus led to a significant reduction of adherent leukocytes in the LDRT-treated group up to 48 h post irradiation and reduced rolling of leukocytes persisted even until 72 h after LDRT [50]. Mice suffering from polyarthritis that were locally treated with a single dose of 0.5 Gy showed decreased inflammatory areas with reduced bone erosions in irradiated feet. Interestingly, the reduction in inflammatory areas was found to be systemic, while effects on bone were only present in the treated foot [48]. Modulation of inflammatory processes was further found in a model of OA, where immune cells in the peripheral blood were altered after local LDRT with 1 × 0.5 Gy alongside an anti-inflammatory shift of cytokines in the serum. More pronounced effects were found in the bone marrow of the mice, where especially a shift from rather inflammatory CD8+ T cells to rather anti-inflammatory CD4+ T cells was observed in both the treated and the untreated leg [18]. A more extensive description of preclinical results has been recently summarized by Rödel et al. [30].

As most of the above-described effects follow a discontinuous dose dependency (meaning there is no direct correlation between the applied dose and the observed effects), the question of which dose is the “optimal dose” for LDRT treatment arises. However, this question cannot easily be answered, as the obtained results often vary depending on various factors.

**Fig. 3:**
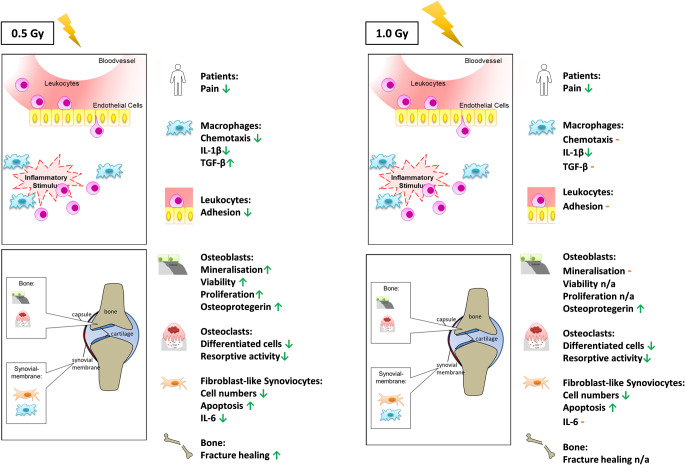
Comparison of the known modulatory effects of low-dose radiation (LDRT; *yellow lightning bolt*) with 0.5 and 1.0 Gy. When comparing 0.5 and 1.0 Gy, no significant differences in patient pain perception has been described, while some differences can be observed in cells of the immune system and the joint. In terms of dose reduction, 0.5 Gy should be the preferred dose in patient treatment. Further, the most beneficial anti-inflammatory and bone-preserving effects are observed at 0.5 Gy. Anti-inflammatory effects are indicated with *green arrows*, experiments that showed no effects are indicated with *orange minus*, while experiments that have not been carried out are indicated as such. *n/a* not available

In that matter, cells that were obtained from healthy or inflamed mice often show different responses to LDRT [48, 51]. Likewise, cells in in vitro experiments also often show different responses to LDRT, depending on whether or not they have been stimulated with inflammatory lipopolysaccharide (LPS) or TNFα, as seen, for example, in the study of Eckert et al. [43]. This suggests that an inflammatory stimulus or milieu is needed in order to achieve the desired anti-inflammatory effects, while in a healthy setting, no or reduced effects are seen [37, 43, 48, 51]. Furthermore, a more physiological condition often seems to be advantageous, as, for example, no alteration in MPH phenotypes was found when looking at them in a single-cell setting, but when cultured together with FLS supernatants, alterations were visible [46]. Likewise, use of laminar flow yields more pronounced effects in EC experiments [43] and 3D cultures show different results to 2D cultures [52]. In line with the inflammatory background, radiosensitivity also seems to play an important role, although doses are relatively low. In that matter, as shown by Frischholz et al., differentiated effects in MPHs were seen depending on the underlying radiosensitivity of the mouse model [44]. In general, there is a discontinuous dose–effect relationship, suggesting potential adjustment the doses used in the clinic depending on the disease. Nevertheless, especially in inflammatory primed diseases of the joints, strong evidence is accumulating for a dose of 0.5 Gy to be especially effective (as summarized in Fig. 3).

## What is the optimal dose for patient treatment?

When determining the optimal treatment dose, two factors should be taken into consideration: which dosage benefits patients best in terms of pain management, and which dose is optimal from a biological point of view. As mentioned above and shown in the Erlangen dose-optimization studies [14, 53, 54] and in patients suffering from OA [18], no significant benefits for 1.0 Gy versus 0.5 Gy were found. In accordance with the clinical results, biological data showed anti-inflammatory effects at doses of 0.5 Gy, with no linear dose–effect correlations. Conferring to present ex vivo and in vivo data, a discontinuous dose–effect correlation has always to be expected in the low and intermediate dose range [40]. This also means that true placebo-controlled studies are necessary in the future, as a lower dose can result in contradictory results in comparison to standard doses.

The number and activity of bone-resorbing OCs were found to be significantly decreased starting at a dose of 0.5 Gy, while bone-forming osteoblasts showed a significant increase in mineralization at 0.5 Gy only. Further, doses of 0.5 and 1.0 Gy both showed a significant increase in the OC-regulating factor osteoprotegerin [48]. These results are in line with findings showing that a dose of 0.5 Gy resulted in increased proliferation and viability of an osteoblast cell line, alongside more rapid fracture healing in an animal model [55]. Next to the effects on bone, FLS were shown to have increased numbers of apoptotic cells starting at 0.5 Gy [48], which points towards a rather anti-inflammatory response. Moreover, MPHs showed an increase in chemotaxis at 0.1 and 0.5 Gy, possibly also contributing to anti-inflammatory mechanisms, as also inflammatory cytokine IL-1β was found to be reduced at 0.5 Gy while rather anti-inflammatory TGF‑β was increased at that dose [56]. This is similar to results in FLS, where inflammatory IL‑6 was significantly reduced at 0.5 Gy [48]. Additionally, leukocyte adhesion was significantly reduced at only 0.5 Gy [57].

All these findings point to an anti-inflammatory and bone-protective mode of action of LDRT (Fig. 3) at 0.5 Gy. While higher doses often also showed some of these anti-inflammatory effects [48], in terms of patient safety in treatment of inflammatory degenerative disorders, a dose reduction is always advised.

## How should biological findings be translated into the clinic?

LDRT constitutes a well-tolerated and efficient treatment option providing long-lasting pain relief and should be offered to OA patients suffering from different conditions of this debilitating disease. However, as described above, while preclinical data are accumulating, placebo-controlled patient studies are necessary to further elucidate the mechanisms underlying LDRT. Next to molecular parameters, potentially influencing factors such as the number and timing of series, patient age, and the type of disease should be investigated by also taking into consideration the sex and inflammatory status. Further, in accordance with preclinical and clinical investigations, a dose of 0.5 Gy seems to be the most beneficial in terms of pain management and molecular effects in inflammatory diseases. A standardization of protocols may further aid in comparing treatment effectiveness and ultimately improve treatment outcome.
